# A low-carbohydrate ketogenic diet promotes ganglioside synthesis via the transcriptional regulation of ganglioside metabolism-related genes

**DOI:** 10.1038/s41598-019-43952-7

**Published:** 2019-05-20

**Authors:** Tetsuya Okuda

**Affiliations:** Bio-Design Research Group, Bioproduction Research Institute, National Institute of Advanced Industrial Science and Technology (AIST), Tsukuba, Japan

**Keywords:** Nutrition, Genetics research

## Abstract

Low-carbohydrate ketogenic diets (LCKDs) are used for treating obesity and epilepsy; however, the molecular mechanism of LCKDs in tissues has not been fully investigated. In this study, novel LCKD-associated molecular targets were explored using gene expression profiling in the liver of mice fed a LCKD. The result showed that the LCKD promoted the expression of glycosyltransferase genes involved in ganglioside synthesis and suppressed the expression of *Gm2a*, the gene encoding GM2 ganglioside activator protein, a lysosomal protein indispensable for ganglioside degradation. These changes were correlated with increased ganglioside content in the liver and serum. As gangliosides are mainly expressed in central nervous tissues, we also analyzed LCKD effect on cerebral cortex. Although ganglioside levels were unchanged in mice on the LCKD, *Gm2a* expression was significantly down-regulated. Further analyses suggested that the LCKD altered the expression levels of gangliosides in a limited area of central nervous system tissues susceptible to *Gm2a*.

## Introduction

In high-fat, low-carbohydrate ketogenic diets (LCKDs), glucose intake is limited, and fat is preferentially utilized by the body as the main energy source. This is associated with favorable effects with respect to lifestyle-related diseases, resulting in improvement of hyperglycemia and suppression of excessive insulin secretion^1,2^. Research using animal models has revealed that the underlying molecular mechanism of LCKDs involves changes in the metabolism of amino acids^3^, carbohydrates^4^, and lipids^2,4–8^ in the liver. Using hyperphagic obese (B6.Cg-*Lep*^*ob*^/J) mice, we established a LCKD feeding model suitable for evaluating the effects of the diet and reported that the LCKD alters the expression of various liver proteins^2,4^ and lipids^2,8^.

**Figure 1:**
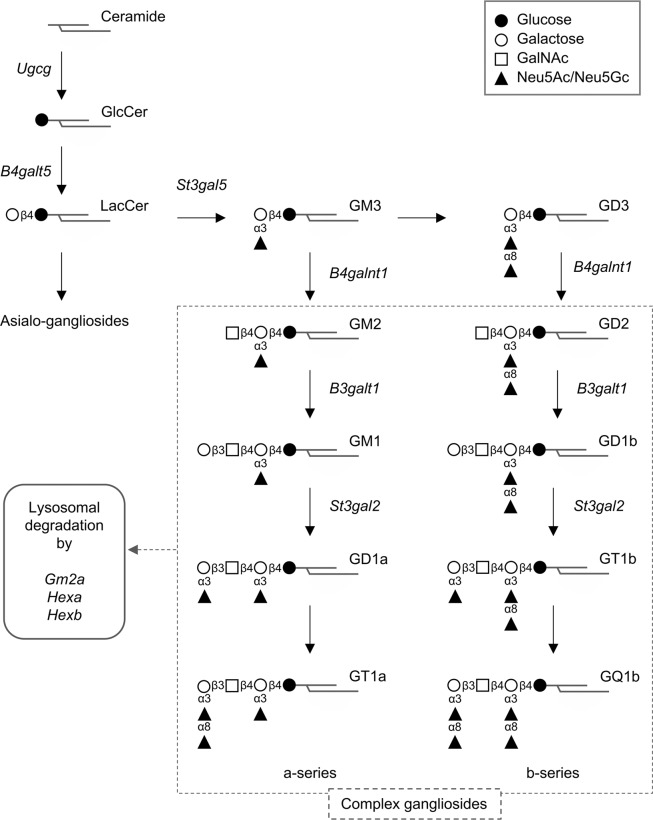
Schematic illustration of the mammalian ganglioside synthesis pathway. Genes encoding ganglioside metabolism-related proteins analyzed in this study are shown in *italics*.

Gangliosides are glycosphingolipids containing sialic acids in the oligosaccharide (Fig. 1). Mammalian cells produce a wide variety of gangliosides that are characterized by their oligosaccharide structure and vary by cell species^9^. Monosialoganglioside GM3 is a major component of the mammalian ganglioside backbone, and a recent study revealed that humans harboring a mutation in *St3gal5* gene encoding α2,3-sialyltransferase 5/GM3 synthase, a key enzyme in GM3 synthesis, develop autosomal recessive infantile-onset symptomatic epilepsy syndrome^10^. Hereditary spastic paraplegia is known to be associated with mutations in the *B4galnt1* gene encoding β1,4-*N*-acetylgalactosyltransferase 1/GM2 synthase, a rate-limiting enzyme in the synthesis of gangliosides with long oligosaccharides (complex gangliosides) that are abundant in central nervous system tissues^11^. These findings indicate that deficiencies in complex gangliosides synthesized by GM3 synthase and GM2 synthase can trigger the onset of epileptic seizure disorder. In addition, abnormal insulin signaling has been observed in mutant mice with GM3 synthase and ganglioside deficiencies^12–14^, indicating that gangliosides modulate insulin signaling *in vivo*.

In the present study, we examined the underlying molecular basis of LCKDs by profiling gene expression in the liver using our established LCKD (F3666, a particular LCKD for rodents)-fed mice. We found that the LCKD affected the expression of ganglioside metabolism-related genes. The LCKD promoted the expression of ganglioside glycosyltransferase genes, including *St3gal5* and *B4galnt1*, and suppressed the expression of *Gm2a*, which encodes the GM2 ganglioside activator protein (GM2AP), a lysosomal protein necessary for the degradation of complex gangliosides^15–17^. These changes in gene expression were correlated with ganglioside content in the liver and serum and resulted in an increase in ganglioside levels. We also analyzed the effects of the LCKD on gene expression in the cerebral cortex, a major tissue expressing gangliosides, and found a significant decrease in *Gm2a* expression. Although the change was not associated with ganglioside levels in cerebral cortex, this result raised a possibility that the diet altered ganglioside metabolism in a limited area of central nervous system tissues.

## Results

### Overall effects of the LCKD on expression of hepatic genes

B6.Cg-*Lep*^*ob*^/J mice fed a LCKD^2^ were used in this study. Our initial purpose was to analyze the effect of LCKD on improvement of the hyperglycemic phenotype of this mice at molecular levels^2^. As this mouse is known to apparently exhibit hyperglycemia in around 10 weeks old under being fed a diet of regular chow^18^, we used this duration (5–12 weeks) for the dietary experiment. We found that LCKD feeding efficiently improved hyperglycemic phenotype of female B6.Cg-*Lep*^*ob*^/J mouse during this duration^2^, thus we determined these as the experimental condition for this study. The primary phenotype in this model was improvement in the hyperglycemic phenotype in the B6.Cg-*Lep*^*ob*^/J mice and production of ketone bodies (β-hydroxybutyrate), whereas dietary intake and body weight gain were unchanged between chow-fed and LCKD-fed mice^2^. Supplementary Figures S1 and S2 detail the phenotypes of the mice used in this study, and all mice were confirmed as exhibiting these phenotypes. Hepatomegaly due to the accumulation of neutral fats, as observed in chow-fed mice, was suppressed in LCKD-fed mice as reported previously^2,8^. Compared to chow-fed mice, the liver weight and total amount of triglycerides decreased by less than 25% in LCKD-fed mice.

**Figure 2:**
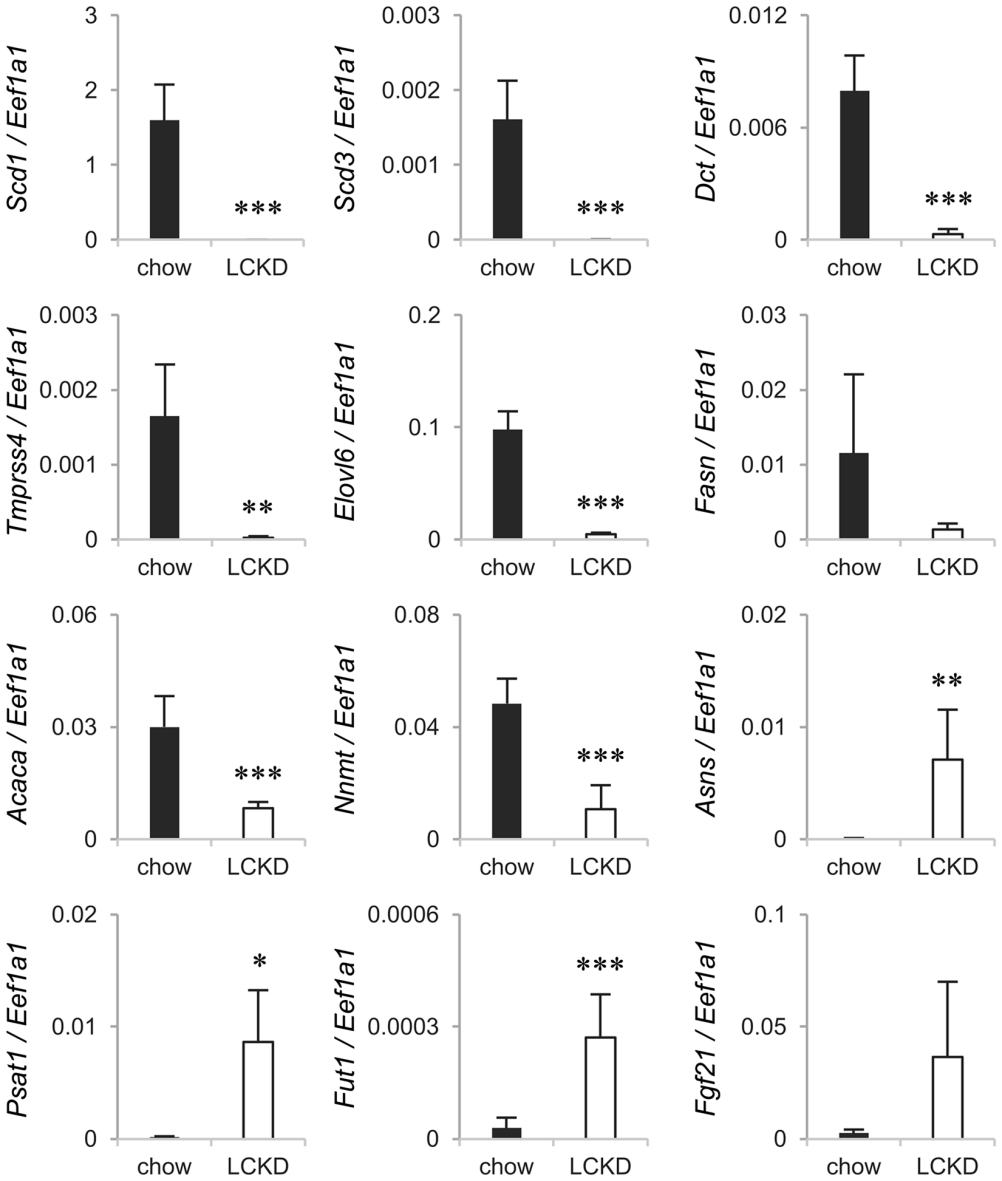
Real-time PCR analysis of the expression of major hepatic genes in LCKD-fed mice. Expression levels of the major hepatic genes that were found to be altered in LCKD-fed mice in this study (Table 1) and previous studies were analyzed by real-time PCR. Expression levels of target genes are shown as the ratio to the expression of the *Eef1a1* gene as an internal control. Closed bars, chow-fed group; open bars, LCKD-fed group. Error bars, mean ± S.D. (n = 6). *P < 0.05, **P < 0.01, ***P < 0.001 chow-fed group vs. LCKD-fed group.

The liver was collected after 7 continuous weeks on the LCKD, and the expression levels of hepatic genes were comprehensively analyzed using an Agilent Expression Microarray. The data for all genes detected as certain signals were compared with data for mice fed regular chow (n = 3) and deposited in the NCBI Gene Expression Omnibus (GEO, http://www.ncbi.nlm.nih.gov/geo/) under accession number GSE115342. The microarray analyses indicated that the LCKD induced a drastic change in hepatic gene expression. Genes exhibiting a marked change in expression (log^2^ ratio > 4 and <−4, *P* < 0.01) are listed in Table 1. Changes in the expression of these extracted genes were verified using real-time PCR with a greater number of samples (n = 6), and genes for which expression was strongly affected by the LCKD were identified (Fig. 2). The genes most strongly affected were those encoding *de novo* lipogenic enzymes for triglyceride synthesis, which previous research showed to be suppressed by LCKDs^5,7,19^. Among these genes, the expression of *Scd1* and *Scd3*, which encode fatty acid desaturases, and *Elovl6*, which encodes a fatty acid elongase, was strongly suppressed by the LCKD. In addition to the genes extracted from the microarray results, real-time PCR analyses also confirmed a significant suppression of expression of the *Acaca* gene, which encodes the rate-determining enzyme in the fatty acid synthesis pathway, and a tendency toward suppression of expression of *Fasn*, which encodes fatty acid synthase. Decreased levels of posttranslational products for these genes have been reported in this LCKD model^2,8^. As these lipogenic genes are targets of insulin^20^ and the LCKD decreases blood insulin levels, the down-regulation of these genes is primarily due to the LCKD-induced decrease in insulin levels. Expression levels of the *Nnmt* and *Asns* genes are also known to be affected by the LCKD^3,21^, and this was also observed in this analysis. The LCKD down-regulates the expression of *Nnmt*, which encodes nicotinamide methyltransferase, and up-regulates *Asns*, which encodes asparagine synthetase. Blood levels of liver-secreted hormone encoded by *Fgf21* also increase in animals fed a LCKD^5–7,22^, and a tendency toward up-regulation in LCKD-fed mice was indicated in the present study by real-time PCR analysis.

**Table 1:** Results of Agilent Expression Microarray analysis of hepatic gene expression in LCKD-fed mice.

Gene	Description	Log^2^ Ratio (LCKD/chow)	P-value
**Major genes**			
*Scd1*	stearoyl-CoA desaturase 1	−10.32	0.000001
*Scd3*	stearoyl-CoA desaturase 3	−8.9	0.002
*Dct*	dopachrome tautomerase	−4.99	0.00003
*Tmprss4*	transmembrane protease serine 4	−4.88	0.000001
*Elovl6*	fatty acid elongase 6	−4.16	0.000007
*Fut1*	fucosyltransferase 1	4.8	0.001
*Psat1*	phosphoserine aminotransferase	6.02	0.006
*Asns*	asparagine synthetase	6.79	0.006
**Genes for ganglioside metabolism**			
*Gm2a*	GM2 ganglioside activator protein	−1.28	0.0005
*Ugcg*	Glucosylceramide synthase	1.04	0.02
*St3gal5*	GM3 synthase	0.56	0.004
*B4galnt1*	GM2 synthase	0.22	0.04
*St3gal2*	GD1a synthase	1.64	0.01

Upper panel shows major genes for which expression strongly changed in mice fed a LCKD (log^2^ ratio > 4 and <−4, *P* < 0.01). Lower panel shows genes involved in ganglioside metabolism for which expression significantly changed (*P* < 0.05).

In the present study, we identified several novel genes for which expression is strongly affected by the LCKD. Expression of *Tmprss4*, which encodes a serine protease (transmembrane protease, serine 4)^23^, and *Dct*, which encodes the enzyme dopachrome tautomerase involved in tyrosine metabolism and melanin synthesis^24^, was strongly suppressed by the LCKD. By contrast, expression of *Psat1*, which encodes serine aminotransferase, and *Fut1*, which encodes fucosyltransferase 1 involved in synthesis of the blood group H (O) antigen^25^, was strongly enhanced by the LCKD. The *Tmprss4*, *Dct*, and *Psat1* genes are thought to regulate cellular levels of the glycogenic amino acids serine and tyrosine and contribute to gluconeogenesis in the liver of LCKD-fed mice. Fucosyltransferase 1 catalyzes the α1,2-fucosylation of the oligosaccharides of glycosphingolipids and glycoproteins and generates the blood group H (O) antigen^25,26^. As fucosylated glycosphingolipids were not detected in the present study, *Fut1* might be involved in the synthesis of fucosylated glycoproteins in the liver.

### Effects of the LCKD on expression of ganglioside metabolism-related genes in the liver

**Figure 3:**
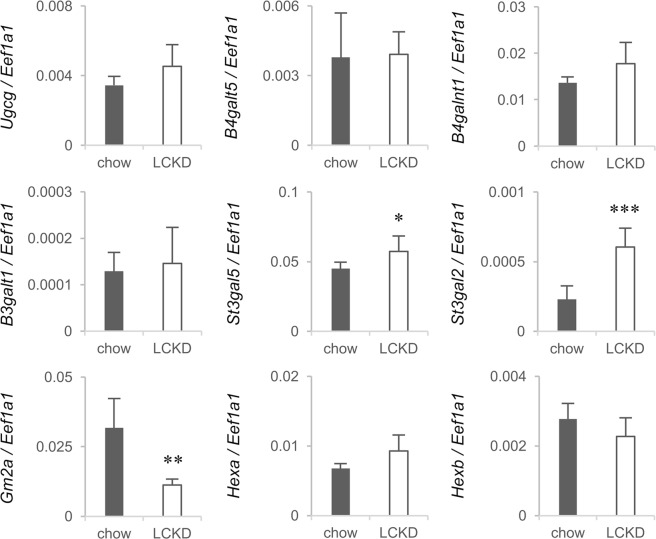
Real-time PCR analysis of the expression of ganglioside metabolism-related genes in the liver of mice fed regular chow or a LCKD. Expression levels of target genes are shown as the ratio to the expression of *Eef1a1* as an internal control. Closed bars, chow-fed group; open bars, LCKD-fed group. Error bars, mean ± S.D. (n = 6). *P < 0.05, **P < 0.01, ***P < 0.001 chow-fed group vs. LCKD-fed group.

We noted that among the genes extracted by the microarray analysis, LCKD affected the expression levels of various ganglioside metabolism-related genes, including *St3gal5* and *B4galnt1*. Mutations in *St3gal5* and *B4galnt1* are associated with inherited epileptic seizure disorders in humans^10,11^, suggesting that these genes play roles in the LCKD-associated suppression of epilepsy^1^. We therefore focused on changes in the expression of ganglioside metabolism-related genes in this study. Microarray analyses showed that the LCKD significantly up-regulated the expression of the ganglioside glycosyltransferase genes *Ugcg*, *St3gal5*, *B4galnt1*, and *St3gal2*, which encode ceramide glucosyltransferase^27^, GM3 synthase^28–30^, GM2 synthase^31^, and α2,3-sialyltransferase 2/GD1a synthase^32^, respectively (Table 1). By contrast, LCKD suppressed the expression of *Gm2a*, which encodes GM2AP^15,16^, a lysosomal protein indispensable for complex ganglioside degradation. GM2AP degrades complex gangliosides in lysosomes in cooperation with β-hexosaminidase A, a product of the *Hexa* and *Hexb* genes^17^. Figure 1 illustrates how these genes are involved in ganglioside metabolism in mammalian cells. Real-time PCR analyses to confirm the microarray results with a greater number of samples (Fig. 3) revealed significant up-regulation of the expression of *St3gal5* and *St3gal2* and down-regulation of *Gm2a*. The expression levels of *St3gal2* and *Gm2a* also changed significantly, even when assessed as *Actb* or *Gapdh* ratios. Real-time PCR analysis of the expression levels of *B4galt5*, *B3galt1*, *Hexa*, and *Hexb*, which are involved in the metabolism of gangliosides (Fig. 1), revealed no changes.

### Effect of the LCKD on ganglioside content in the liver and serum

**Figure 4:**
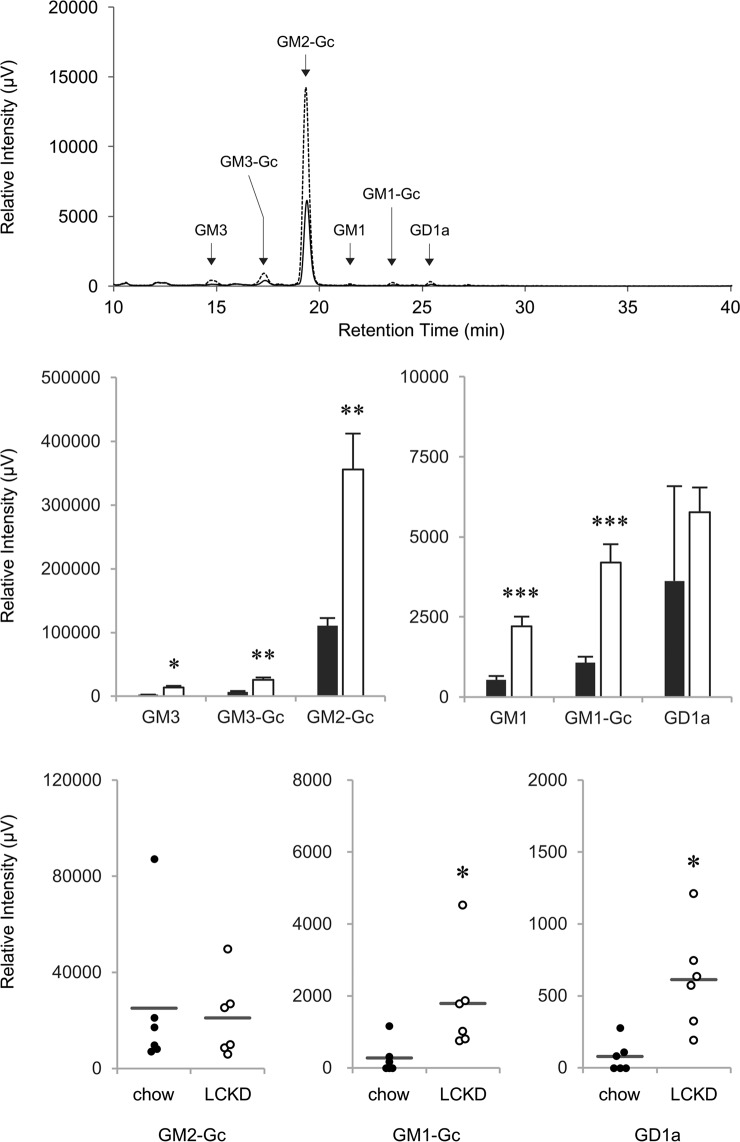
HPLC analysis of ganglioside levels in liver and serum of LCKD-fed mice. Representative HPLC chromatogram of gangliosides purified from the liver using an oligosaccharide fluorescent labeling method (upper panel). Solid line, gangliosides from chow-fed group; dotted line, gangliosides from LCKD-fed group. Samples equivalent to gangliosides purified from 1.5 mg of tissue were analyzed. The elution times of standard 2-AA oligosaccharides are indicated by arrows. Relative levels of gangliosides in mouse liver were calculated based on the peak area (μV∙sec) of each 2-AA oligosaccharide in the HPLC analysis (middle panels). Closed bars, chow-fed group; open bars, LCKD-fed group. Error bars, mean ± S.D. (n = 3). *P < 0.05, **P < 0.01, ***P < 0.001 chow-fed group vs. LCKD-fed group. Relative levels of gangliosides in mouse serum were determined based on the peak area (μV∙sec) of each 2-AA oligosaccharide in the HPLC analysis (lower panels). Data from individual samples are plotted. Closed circles, chow-fed group; open circles, LCKD-fed group. Error bars, mean ± S.D. (n = 6). *P < 0.05 chow-fed group vs. LCKD-fed group.

Changes in the expression levels of *St3gal5*, *St3gal2* and *Gm2a* were suggestive of increased expression of liver gangliosides. Our previous results obtained by thin-layer chromatography of hepatic lipids in this model mouse showed LCKD effect on the expression of the liver gangliosides^8^, which supported this idea. However, ganglioside has diversity in its oligosaccharide structure, and a number of genes regulate the structural diversity of gangliosides. Previously, we used crude preparations of liver gangliosides for analysis^8^. Thus, we couldn’t verify the relationship between expression change of each genes and corresponding ganglioside synthesis in liver. In the current study, in order to clarify the relationship, we performed HPLC analysis to show more detail alteration of each molecular species of liver gangliosides. Thus, we analyzed hepatic gangliosides using a semi-quantitative HPLC approach with an oligosaccharide fluorescent labeling method (Fig. 4). In this analysis, the ceramide portion of the gangliosides was removed using *Rhodococcus triatomea* endoglycoceramidase I (EGCase I)^33^, which catalyzes the hydrolysis of the β-glycosidic linkage between oligosaccharides and ceramides in various glycosphingolipids. The released oligosaccharides were then sequentially labeled with anthranilic acid (2-AA). The 2-AA-labeled oligosaccharides were analyzed by HPLC, with the fluorescence intensity of the 2-AA serving as a measure of the amount of each ganglioside. These analyses indicated that the gangliosides contained GM2 as the primary component and GM3, GM1, and GD1 as minor components. The gangliosides primarily contained Neu5Gc as the sialic acid (shown as GM2-Gc, GM3-Gc, and GM1-Gc), but Neu5Ac was also detected. Collectively, these analyses indicated that the LCKD induced an increase of >3-fold in the expression of all hepatic gangliosides except GD1a. As gangliosides produced in the liver are secreted into the blood, we also analyzed the serum ganglioside content by HPLC. GM2-Gc, GM1-Gc, and GD1a were detected in the serum, and levels of GM1-Gc and GD1a were significantly increased in mice fed the LCKD. These results indicate that up-regulation of *St3gal5* and *St3gal2* by LCKD promotes synthesis of GM3 and GD1a respectively, and down-regulation of *Gm2a* promotes increase of GM2 and its derivatives in liver.

### Effect of the LCKD on the expression of cerebral cortex genes

**Figure 5:**
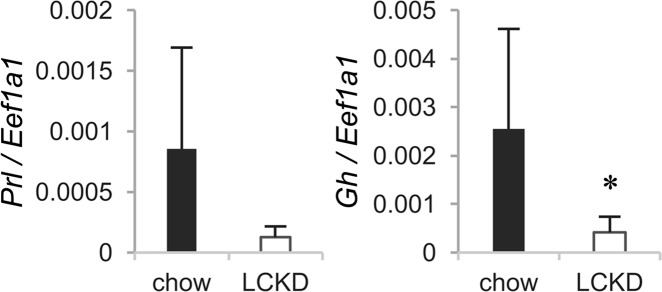
Real-time PCR analysis of the expression of major cerebral cortex genes (Table 2) for which expression changed in microarray analysis of samples from LCKD-fed mice. Expression levels of target genes are shown as the ratio to the expression of *Eef1a1* as an internal control. Closed bars, chow-fed group; open bars, LCKD-fed group. Error bars, mean ± S.D. (n = 6). *P < 0.05 chow-fed group vs. LCKD-fed group.

As gangliosides are mainly expressed in central nervous tissues, we also analyzed the effect of the LCKD on gene expression in the cerebral cortex using the Agilent Expression Microarray. The cerebral cortex was isolated from whole brain as described in the *Methods*. The hippocampal region was left in the cerebral cortex to ensure a certain amount of tissue for ganglioside analysis. The resulting data were deposited in the GEO under accession number GSE115342. The analysis showed that changes in gene expression in the cerebral cortex mediated by the LCKD are relatively small when compared with those in the liver. Obvious changes (log^2^ ratio < −4, *P* < 0.01) were observed only for the genes *Prl* and *Gh* (Table 2), which encode the pituitary hormones prolactin and growth hormone, respectively, and are known to be expressed in the cerebral cortex^34,35^. Several studies have reported a relationship between the expression of these genes and epilepsy^35,36^. In validation analyses using real-time PCR, a significant decrease was detected in the expression of *Gh*, and a decreasing trend was observed in the expression of *Prl* (Fig. 5).

**Table 2:** Results of Agilent Expression Microarray analysis of cerebral cortex gene expression in LCKD-fed mice.

Gene	Description	Log^2^ Ratio (LCKD/chow)	P-value
*Prl*	prolactin	−4.6	0.01
*Gh*	growth hormone	−4.4	0.01

Data show major genes for which expression strongly changed in mice fed a LCKD (log^2^ ratio > 4 and <−4, *P* < 0.01).

**Figure 6:**
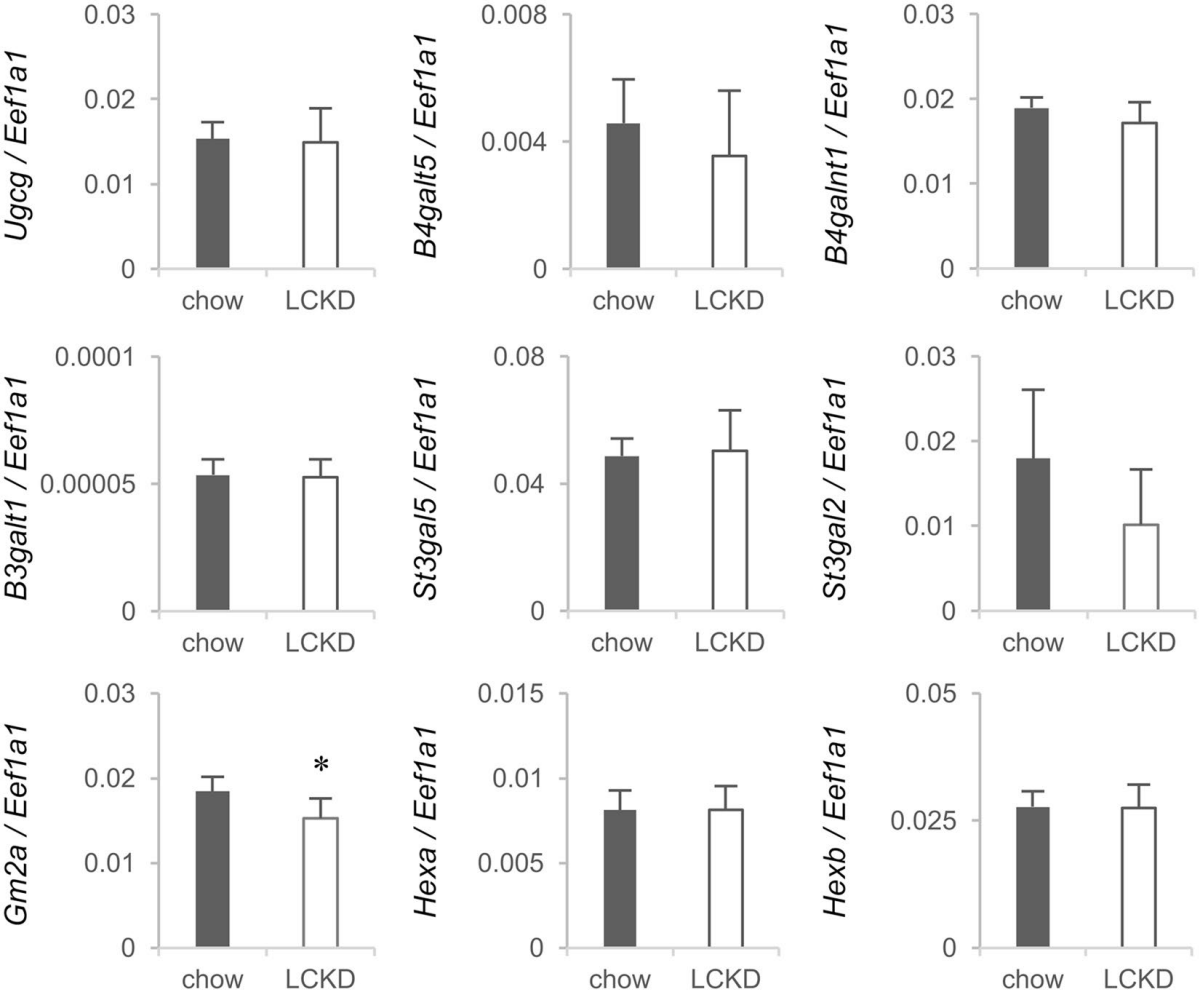
Real-time PCR analysis of ganglioside metabolism-related gene expression in the cerebral cortex of mice fed regular chow or a LCKD. Expression levels of target genes are shown as the ratio to the expression of *Eef1a1* as an internal control. Closed bars, chow-fed group; open bars, LCKD-fed group. Error bars, mean ± S.D. (n = 6). *P < 0.05 chow-fed group vs. LCKD-fed group.

Regarding ganglioside metabolism-related genes, no obvious changes were observed in the microarray analysis, but a significant decrease in *Gm2a* expression was detected by real-time PCR analysis (Fig. 6). However, an effect of the LCKD on ganglioside content in the cerebral cortex was not observed in the HPLC analysis. The elution patterns of cerebral cortex gangliosides in the HPLC analysis are shown in Supplementary Fig. S3. The cerebral cortex contains GM1, GD1a, GD1b, and GT1b as major components and GM3, GM2, GT1a, and GQ1b as minor components. The gangliosides contained Neu5Ac as the sialic acid, but Neu5Gc was barely detectable. There were no significant differences between mice fed regular chow and those fed the LCKD.

## Discussion

This study revealed that a LCKD promotes the expression of genes involved in the synthesis of liver gangliosides and suppress the expression of *Gm2a*, which is involved in the degradation of GM2 and complex gangliosides. These changes are correlated with an increase in ganglioside content in the liver, indicating that LCKDs promote ganglioside synthesis in tissues via the transcriptional regulation of ganglioside metabolism-related genes. Our previous data^8^ had been suggested that LCKD effect on the synthesis of liver gangliosides, and this study using gene expression profiling and HPLC ganglioside analysis revealed that details of mechanism of LCKD effect on the synthesis of liver gangliosides. In particular, the finding that ganglioside synthesis can be regulated by the gene expression level of its degradation factor, is conceptually new in tissue ganglioside metabolism.

We previously showed that the expression of gangliosides is promoted by treating cells with a glucose starvation mimetic^37^. This suggests that the decreased carbohydrate intake associated with LCKDs induces an increase in hepatic ganglioside expression. Although we analyzed the mechanism of transcriptional regulation by this mimetic^37–40^, no clear answer was obtained. It will therefore be necessary to employ a new approach based on the results of the present study. The LCKD-fed mice model using this study was designed for analysis of effect of low glucose supply to tissues by LCKD^2^. Thus, the further analysis using this model is a valid approach to the mechanism. Although we did observe changes in the expression levels of insulin target genes in this LCKD model, a number of previous studies have not found an effect of insulin on the transcription of ganglioside synthase genes.

Gangliosides are known to modulate the function of membrane proteins^9^, and deficiency or excessive accumulation of gangliosides can lead to several pathologic conditions, including oncogenic transformation^41^, insulin resistance^42^, obesity^43^, and neurologic diseases^17,44^. An appropriate amount of ganglioside is necessary for maintaining the integrity of cellular membranes^43^; thus, a modest increase in levels of tissue gangliosides induced by a LCKD could contribute to cell and tissue soundness.

Increased levels of liver gangliosides GD1a and GM1-Gc were also detected in the serum of mice fed the LCKD. As no significant increase in GD1a level was detected in the liver, highly hydrophilic gangliosides with long oligosaccharides could be secreted into the plasma from the liver. By contrast, the level of GM2-Gc, the major ganglioside, was increased in the liver of LCKD-fed mice but not significantly increased in the serum, indicating that GM2-Gc tends to remain in the liver. We therefore believe that the plasma ganglioside content can be utilized as an index of the effect of LCKDs on tissue ganglioside metabolism.

We previously found a unique phenotype in LCKD-fed mice: hepatomegaly due to the accumulation of neutral fats, as observed in chow-fed mice, was suppressed in LCKD-fed mice^2^. In chow-fed mice, the weight of the liver increased more than 3-fold as compared with the liver of LCKD-fed mice^2,8^, but both groups exhibited similar gains in body weight (Supplementary Fig. S2). This phenotype is unique in that feeding of LCKD promotes obesity whereas only steatosis is prevented in in the mice. Levels of gangliosides, which are thought to regulate insulin signaling^12–14^, were increased in LCKD-fed mice (Fig. 4), in contrast to triglyceride and cholesterol levels, which decreased^2,8^. One of the causes of triglyceride accumulation in the liver of chow-fed mice is hyper-activation of the *de novo* lipogenic pathway mediated by insulin signaling^2^, and this is also associated with abnormal modifications of insulin signaling modulators^4^. As these abnormalities do not occur in mice fed a LCKD^2,4^, the LCKD-induced increase in ganglioside levels likely acts to normalize insulin signaling and thus suppress the development of fatty liver.

The LCKD had no effect on the expression levels of gangliosides in the cerebral cortex, but down-regulation of *Gm2a* expression was detected, as in the liver. In mice with a *Gm2a* gene disruption, neuronal storage of gangliosides occurs only in restricted regions of the brain, such as the piriform, entorhinal cortex, amygdala, hypothalamic nuclei, and cerebellum^15^. This observation suggests that the LCKD-induced down-regulation of *Gm2a* observed in the present study should lead to increases in the ganglioside content in these areas. To examine this hypothesis, we preliminary investigated ganglioside levels in cerebellum of LCKD-fed mice by HPLC (Supplementary Fig. S4). The result showed a tendency toward higher levels of GD1a and its derivatives in the cerebellum of LCKD-fed mice, which supported our hypothesis. However, GD2 in the same sample showed a decreasing tendency that could not be explained by down-regulation of *Gm2a*. Further investigation will thus be required to fully elucidate the effect of LCKDs on central nervous system tissues.

In conclusion, the results of the present study demonstrate that LCKDs induce increased expression of tissue gangliosides via transcriptional regulation of ganglioside metabolism-related genes. The effects of the diet are reflected in the ganglioside content in liver, serum, and may involve in the limited area of central nervous system tissues susceptible to *Gm2a*.

## Methods

### Animals and dietary studies

Female B6.Cg-*Lep*^*ob*^/J mice (Charles River Laboratories Japan, Yokohama, Japan) were used in this study, and all animals were maintained as reported previously^2^. CE-2 (CLEA Japan, Tokyo, Japan), comprised of 58.2% carbohydrates, 12.6% fat, and 29.2% protein by calories was used as regular chow. F3666 (Bio-Serv, Frenchtown, NJ), comprised of 1.7% carbohydrates, 93.9% fat, and 4.4% protein by calories was used as the LCKD. Five-week-old mice were raised on either regular chow or the LCKD for a period of 7 weeks. During this period, blood glucose and β-hydroxybutyrate levels were monitored at 3:00 pm every 2 weeks, and tissue samples were collected at the end of the dietary study. The blood glucose and β-hydroxybutyrate levels were examined with venous blood collected from the tail vein by using the Precision Xceed Monitoring System (Abbott Laboratories, Abbott Park, IL, USA). For sample collection, mouse was anesthetized with isoflurane and sacrificed by blood drawing from heart. Then, liver, serum, cerebral cortex, and cerebellum were collected, and were immediately frozen in liquid nitrogen and stored at −80 °C until used in experiment. The cerebral cortex, hippocampus and cerebellum were isolated from whole brain, then removed deep gray matter, mid brain, brain stem, and olfactory bulb from the cerebral cortex. The hippocampus was not removed from the cerebral cortex. The blood was incubated at 37 °C for 1.5 hours, then removed clot and centrifuged at 800 *g* for 10 min, and the supernatant was collected as serum. Two mice were maintained with chow or LCKD at the same term respectively, and the collected samples were analyzed at the same experiment. In gene expression analysis, data of samples from independent experiments were normalized by total RNA levels and internal control gene. For ganglioside analysis, data were normalized by the wet weight of the tissue samples, and monitored for no experimental loss by using standard ganglioside as a positive control.

The Committee for Experiments Involving Animals of the National Institute of Advanced Industrial Science and Technology (AIST) approved all animal experiments, and all experiments were performed in accordance with relevant guidelines and regulations.

### Gene expression analysis

Total RNA was isolated from tissues using a NucleoSpin^®^ RNA Midi kit (Macherey-Nagel, Duren, Germany). cDNA was synthesized using the SuperScript^®^ III First-Strand Synthesis System for RT-PCR (Thermo Fisher Scientific, Waltham, MA) from 3 μg of total RNA as a template. Agilent Expression Microarray analysis for gene expression profiling in tissues was conducted by Takara Bio (Shiga, Japan). Briefly, Cy3-labeled cRNA was prepared from 0.1 μg of total RNA using a Low-Input Quick Amp Labeling kit (Agilent Technologies, Santa Clara, CA) according to the manufacturer’s instructions, followed by RNeasy column purification (Qiagen, Valencia, CA). The Cy3-labeled cRNA (0.6 μg) was fragmented at 60 °C for 30 min in a reaction volume of 25 μl containing 1× Agilent fragmentation buffer and 2× Agilent blocking agent, to which 25 μl of 2× Agilent hybridization buffer was added, and the reaction was hybridized to a SurePrint G3 Mouse GE v2 8 × 60 K Microarray (Agilent Technologies) for 17 h at 65 °C in a rotating Agilent hybridization oven. After hybridization, the microarray slide was washed for 1 min at room temperature with Agilent GE wash buffer 1 and 1 min with 37 °C Agilent GE wash buffer 2 and scanned immediately after washing on an Agilent SureScan Microarray Scanner (G2600D) using a one-color scan setting for 8 × 60 K array slides (scan area 61 × 21.6 mm, scan resolution 3 μm, dye channel set to green PMT 100%). Scanned images were analyzed with Agilent Feature Extraction Software 12.0.3.1 using default parameters to obtain background-subtracted and spatially de-trended processed signal intensities. Processed signal intensities were normalized using the global scaling method. The trimmed mean probe intensity was determined by disregarding the upper and lower 2% of the probe intensities in order to calculate the scaling factor. Normalized signal intensities were then calculated from the target intensity of each array using the scaling factor, so that the trimmed mean target intensity of each array was arbitrarily set to 2500. The resulting microarray data were analyzed using the Aqua microarray viewer and Aqua *t*-test (Takara Bio) and deposited in GEO (accession number GSE115342).

Relative quantification of target gene expression by real-time PCR was performed using a Light Cycler^®^ 480 II system (Roche, Penzberg, Germany) with gene-specific primers (summarized in Table S1). Reactions were performed using a KAPA SYBR^®^ FAST qPCR kit (KAPA Biosystems; Wilmington, MA) according to the manufacturer’s instructions. The crossing points were defined as the PCR cycle number at which the fluorescence increased appreciably above background, and the expression levels of target genes were calculated from the crossing points (C_t_ values) on the premise that the amount of target gene amplified doubled in one PCR cycle. Using this system, we first analyzed the expression levels of several housekeeping genes (*Actb*, *Gapdh*, *Eef1a1*) and found that *Eef1a1* was most stably expressed in the liver and cerebral cortex of the mice used in this study (Supplementary Fig. S5). Thus, we used *Eef1a1* as the internal reference gene for real-time PCR analyses.

### Ganglioside extraction and HPLC analysis

Ganglioside extraction was performed as reported previously^37^ with slight modifications. The total lipids from 50–100 mg of tissues or 50 µl of serum were sequentially extracted with chloroform/methanol/water at 2:1:0 and 1:2:0.8 (*v*/*v*/*v*), respectively. Gangliosides were separated by Folch partitioning, and purified using a MonoSpin^®^ C18 column (GL Sciences, Tokyo, Japan).

Ganglioside levels were determined by semi-quantitative HPLC using a glycosphingolipid fluorescent labeling method^37,45^, with slight modifications. For fluorescent labeling, the ceramide moieties of purified gangliosides (corresponding to 30 mg of liver, 10 mg of brain tissue, and 25 µl of serum) were released by incubation in the presence of 6 mU of EGCase I (New England Biolabs, Ipswich, MA) at 37 °C for 16 h, according to the manufacturer’s instructions. The reductive end of the oligosaccharide was fluorescently labeled using 2-AA (Sigma-Aldrich) by incubation in 80 μl of labeling mixture (45 mg/ml 2-AA, 40 mg/ml sodium acetate trihydrate, 20 mg/ml boric acid, and 45 mg/ml sodium cyanoborohydride in methanol) at 80 °C for 1 h. The 2-AA-labeled oligosaccharides were purified using a MonoSpin^®^ Amide column (GL Sciences) and analyzed using a TSK gel-amide 80 column (Tosoh, Tokyo, Japan) with an LC-2000 Plus HPLC system (JASCO, Tokyo, Japan). The chromatography system and fluorescence detection/gradient conditions were identical to those described previously^45^.

### Statistical analysis

After determination of variance by the F-test, statistical significance was determined using the two-tailed Student’s *t*-test, with statistical significance defined as follows: *P < 0.05, **P < 0.01, ***P < 0.001.

## Supplementary information

Supplementary Informations

## Data Availability

The datasets generated during and/or analyzed during the current study are available in the Gene Expression Omnibus (GEO), repository, as accession number GSE115342 (https://www.ncbi.nlm.nih.gov/geo/query/acc.cgi?acc=GSE115342), or from the corresponding author on reasonable request.
