# Supplementary material for: A low-carbohydrate ketogenic diet promotes ganglioside synthesis via the transcriptional regulation of ganglioside metabolism-related genes
Source: Sci Rep. 2019 May 20;9:7627. doi: 10.1038/s41598-019-43952-7 (PMC6527835; doi:10.1038/s41598-019-43952-7)
Supplement: Supplementary file 1 — Supplementary Informations [file 41598_2019_43952_MOESM1_ESM.pdf]

A low-carbohydrate ketogenic diet promotes ganglioside synthesis via the transcriptional regulation of ganglioside metabolism-related genes

Tetsuya Okuda*

Supplementary Information

Supplemental Figure S1
Supplemental Figure S2
Supplemental Figure S3
Supplemental Figure S4
Supplemental Figure S5
Table S1

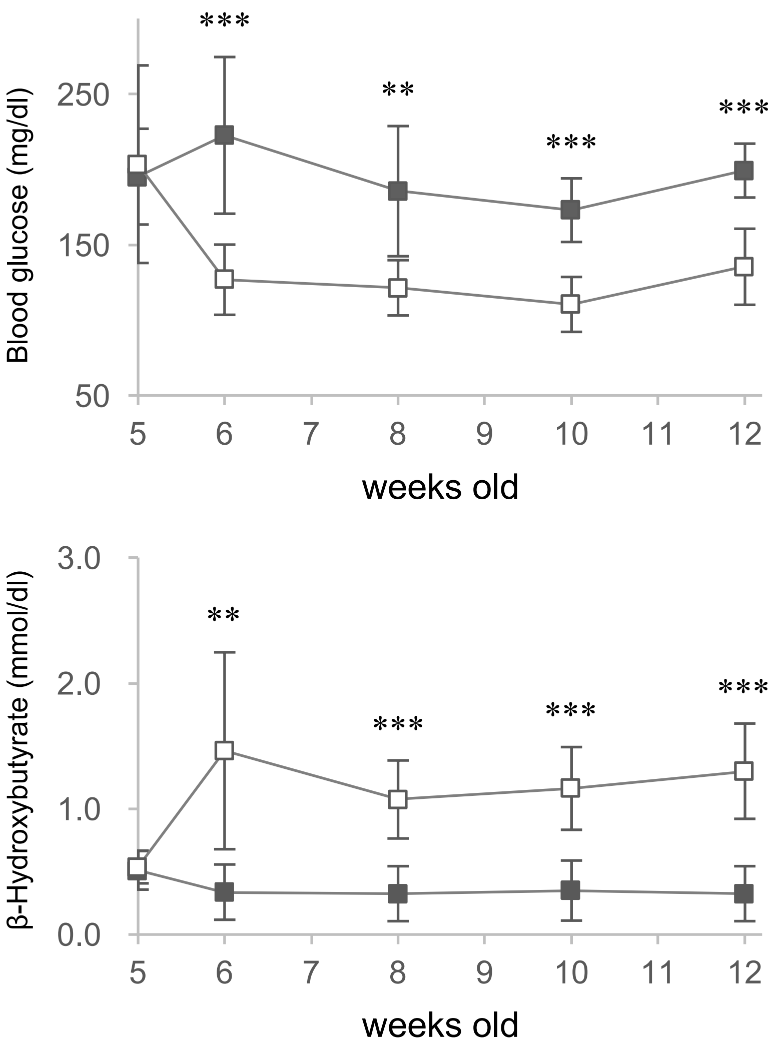

Figure S1. Effect of the LCKD on blood glucose and β-hydroxybutyrate levels. Blood glucose (upper panel) and β-hydroxybutyrate (lower panel) levels in chow-fed and LCKD-fed mice during the experimental period (5 to 12 weeks of age). Filled squares with solid line, chow-fed group; open squares with solid line, LCKD-fed group. Error bars, mean ± S.D. (n=8). ***P*<0.01, ****P*<0.001 chow-fed group vs. LCKD-fed group.

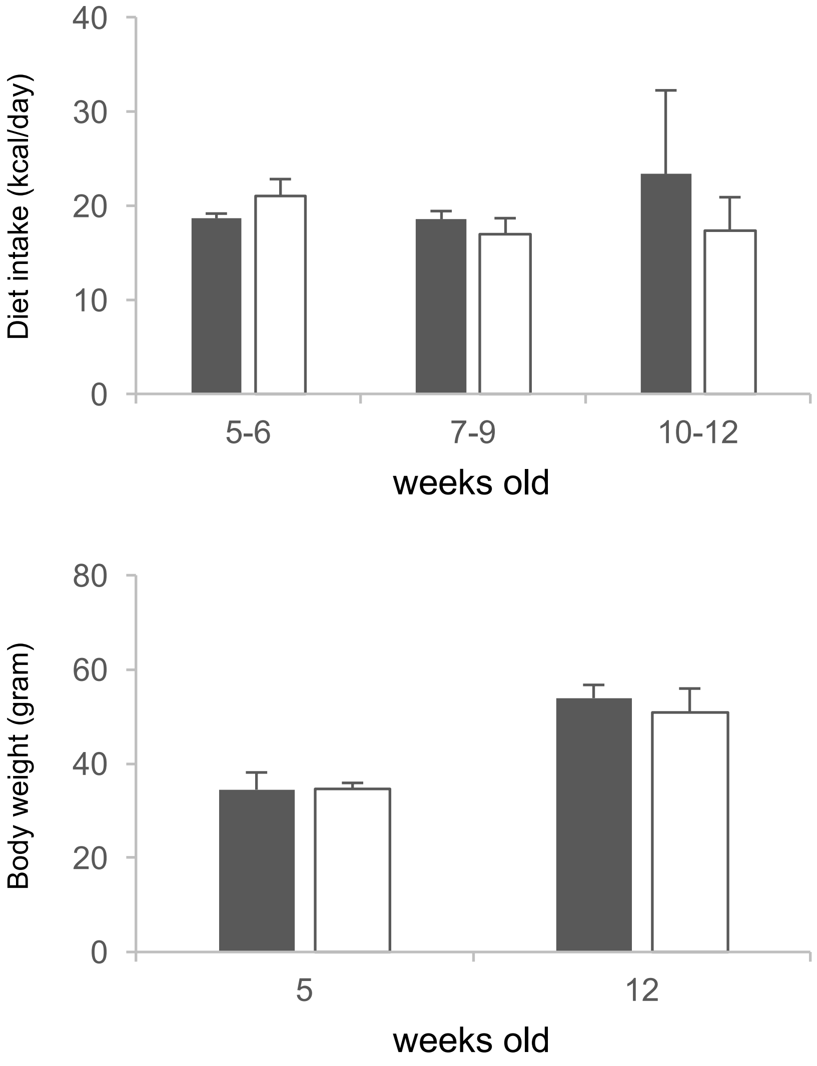

Figure S2. Diet intake and body weight of LCKD-fed mice. Upper panel, average daily diet intake (kcal) in chow-fed (closed bars) and LCKD-fed (open bars) mice during the experimental period. Lower panel, average body weight (grams) of chow-fed and LCKD-fed mice at the beginning and end of the study. Error bars, mean ± S.D. (n=6-8).

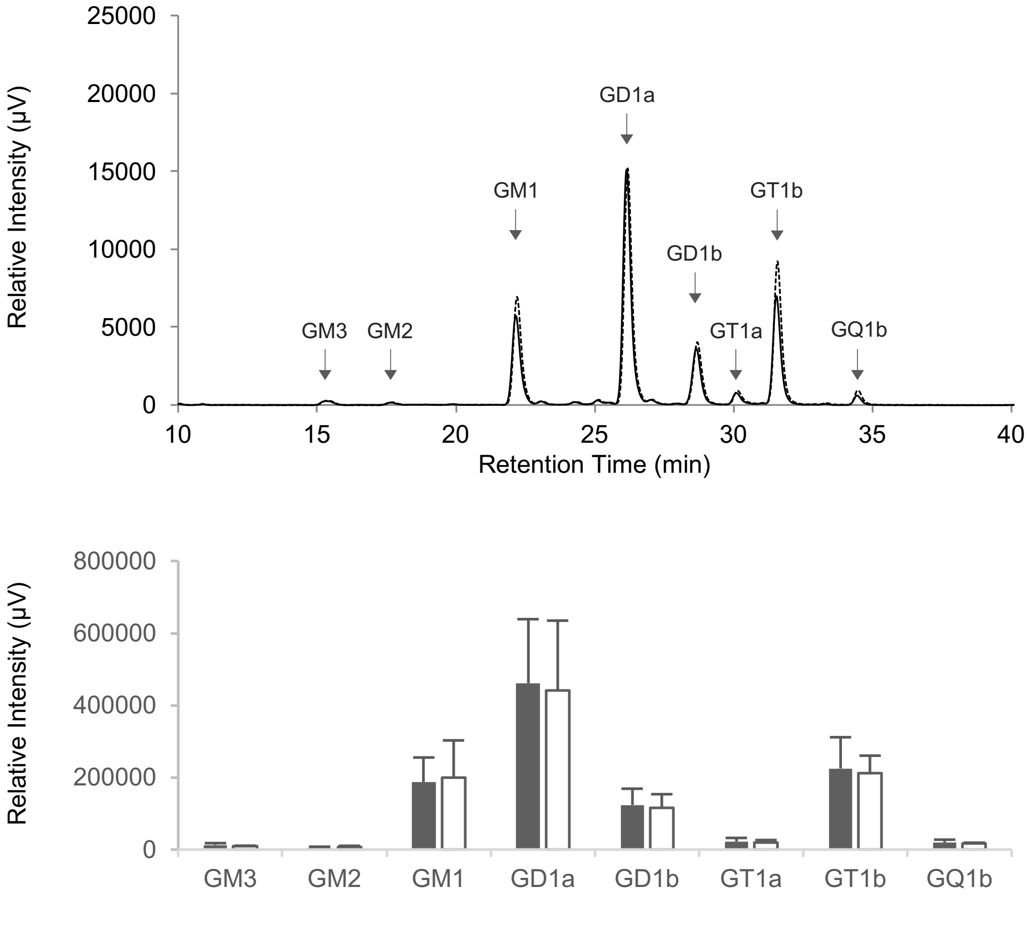

Figure S3. Representative HPLC chromatograms of gangliosides purified from the cerebral cortex of chow-fed (solid line) and LCKD-fed (dotted line) mice (upper panel). An oligosaccharide fluorescent labeling method as described in the Methods was used in the HPLC analysis. Samples equivalent to gangliosides purified from 0.5 mg of tissue were analyzed. The elution times of standard 2-AA oligosaccharides are indicated by arrows. Relative levels of gangliosides in the cerebral cortex were calculated based on the peak area (µV·sec) of each 2-AA oligosaccharide in the HPLC analysis (lower panel). Closed bars, chow-fed group; open bars, LCKD-fed group. Error bars, mean ± S.D. (n=3).

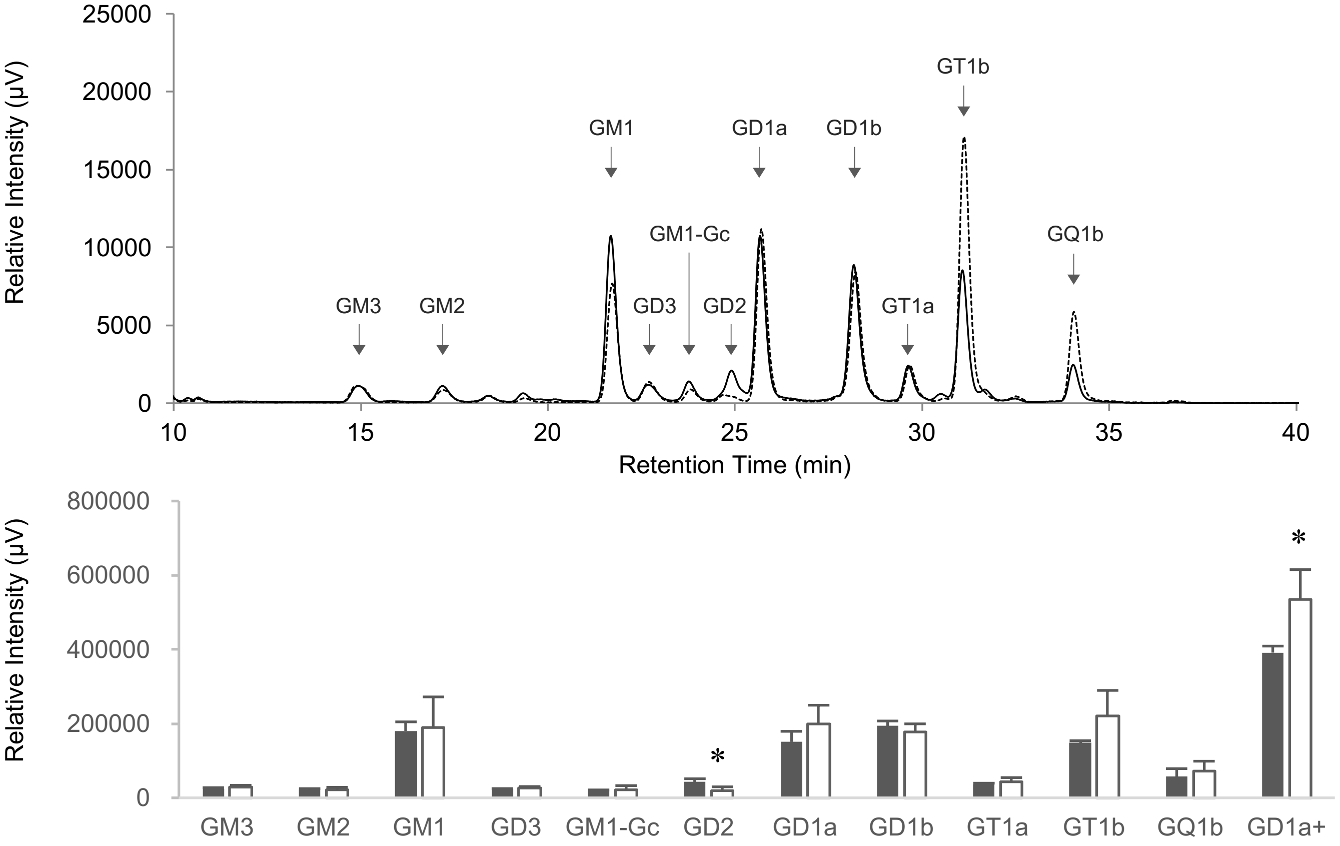

Figure S4. Representative HPLC chromatograms of gangliosides purified from the cerebellum of chow-fed (solid line) and LCKD-fed (dotted line) mice (upper panel). An oligosaccharide fluorescent labeling method as described in the Methods was used in the HPLC analysis. Samples equivalent to gangliosides purified from 0.5 mg of tissue were analyzed. The elution times of standard 2-AA oligosaccharides are indicated by arrows. Relative levels of gangliosides in the cerebellum were calculated based on the peak area (µV·sec) of each 2-AA oligosaccharide in the HPLC analysis (lower panel). “GD1a+” represents all GD1a derivatives (GD1a, GT1a, GT1b, and GQ1b). Closed bars, chow-fed group; open bars, LCKD-fed group. Error bars, mean ± S.D. (n=3). **P*<0.05 chow-fed group vs. LCKD-fed group.

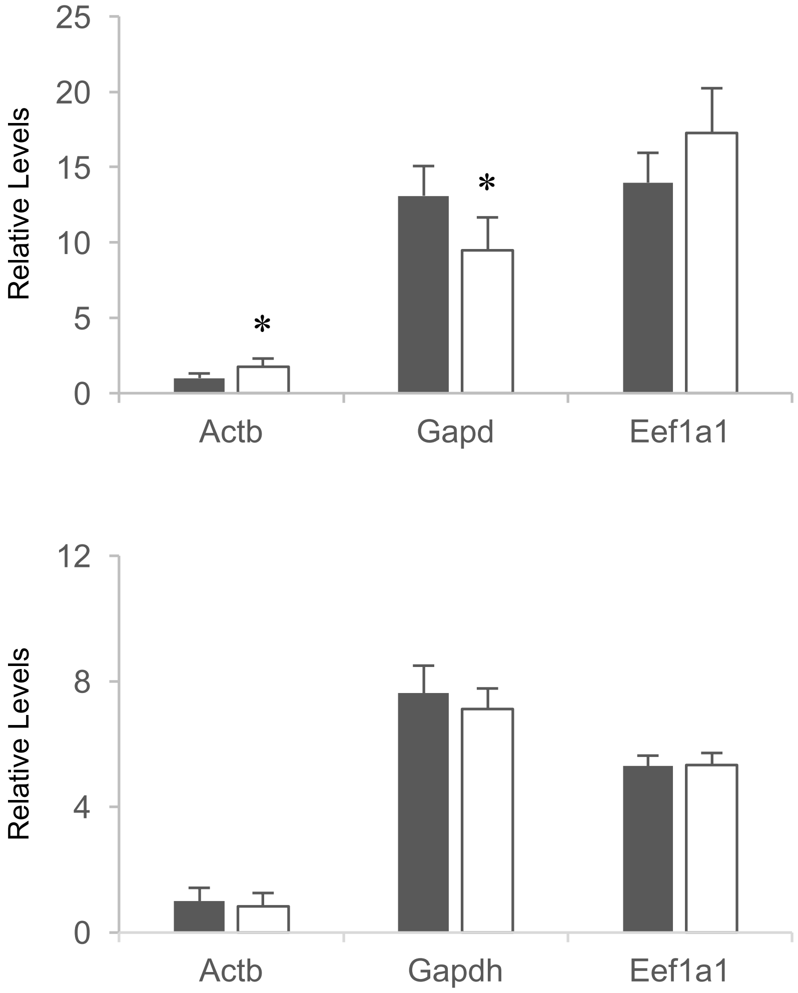

Figure S5. Real-time PCR analysis of the expression levels of internal control genes. Expression levels of housekeeping genes (*Actb*, *Gapdh*, *Eef1a1*) in the liver (upper panel) and brain cortex (lower panel) are shown as the ratio to the expression level of *Actb* in the chow-fed group. Significant differences (**P*<0.05 chow-fed vs. LCKD-fed) were observed only for *Actb* and *Gapd* in the liver. Expression levels were calculated based on the C_t value as described in the Methods. Closed bars, chow-fed group; open bars, LCKD-fed group. Error bars: mean ± S.D. (n=8).

Table S1. Primers used for real-time PCR analyses

Gene (Number¶)	Primer sequence
Actb	Forward: 5’- ctaaggccaaccgtgaaaag
(NM_007393)	Reverse: 5’- accagaggcatacagggaca
Gapdh	Forward: 5’- tgtccgtcgtggatctgac
(NM_008084)	Reverse: 5’- cctgcttcaccaccttcttg
Eef1a1	Forward: 5’- acacgtagattccggcaagt
(NM_010106)	Reverse: 5’- aggagccctttcccatctc
Scd1	Forward: 5’- ttccctcctgcaagctctac
(NM_009127)	Reverse: 5’- cagagcgctggtcatgtagt
Scd3	Forward: 5’- ctgacctgaaagccgagaag
(NM_024450)	Reverse: 5’- gcagaatgccaggcttgta
Dct	Forward: 5’- ggctacaattacgccgttg
(NM_010024)	Reverse: 5’- cactgagagagttgtggaccaa
Tmprss4	Forward: 5’- tcaggtgcagaatggaagc
(NM_145403)	Reverse: 5’- ggctctttccacagtcaagg
Elovl6	Forward: 5’- cagcaaagcacccgaacta
(NM_130450)	Reverse: 5’- aggagcacagtgatgtggtg
Fasn	Forward: 5’- gctgctgttggaagtcagc
(NM_007988)	Reverse: 5’- agtgttcgttcctcggagtg
Acaca	Forward: 5’- gcgtcgggtagatccagtt
(NM_133360)	Reverse: 5’- ctcagtggggcttagctctg
Nnmt	Forward: 5’- tgtgatcttgaaggcaacaga
(NM_010924)	Reverse: 5’- ttgattgcacgcctcaac
Asns	Forward: 5’- ctgcctctgctccacctt
(NM_012055)	Reverse: 5’- cacatgctacaggcggact
Psat1	Forward: 5’- ccggtggatgtttccaag
(NM_001205339)	Reverse: 5’- ggtcatcccggacaatca
Fut1	Forward: 5’- aaagaattcgcttgcaccac
(NM_008051)	Reverse: 5’- gagagcacacagaccaacaga
Fgf21	Forward: 5’- agatggagctctctatggatcg
(NM_020013)	Reverse: 5’- gggcttcagactggtacacat
Ugcg	Forward: 5’- agtttcaatccagaatgatcagg
(NM_011673)	Reverse: 5’- cattctgaaattggctcacaaat

B4galt5	Forward: 5'- gatgacgacttgtggaacagg
(NM_019835)	Reverse: 5'- ttttcctgtgtccccttctg
B4galnt1	Forward: 5'- gcagctgcaactggtgact
(NM_008080)	Reverse: 5'- tggtggagaaccggactg
B3galt1	Forward: 5'- cagctggttgggttccttc
(NM_020283)	Reverse: 5'- tttcttccaaacctgctgga
St3gal5	Forward: 5'- gattctggggccatgataag
(NM_011375)	Reverse: 5'- actgaggtcgtagccaaagc
St3gal2	Forward: 5'- gctctggctatggacaagaagt
(NM_009179)	Reverse: 5'- acagtcggtgcctgattcat
Gm2a	Forward: 5'- ccttgagggcaagaccag
(NM_010299)	Reverse: 5'- tccttctccacggtgagc
Hexa	Forward: 5'- acctgggaggggatgaagt
(NM_010421)	Reverse: 5'- atgaaggcctggatgttgg
Hexb	Forward: 5'- ccattgtttggcaagaagttt
(NM_010422)	Reverse: 5'- ttccacacttcgactactgtgc
Prl	Forward: 5'- gttctctcaggccatcttgg
(NM_011164)	Reverse: 5'- aggaggagtgtccctgcttt
Gh	Forward: 5'- gcttggcaatggctacaga
(NM_008117)	Reverse: 5'- ggaaaagcactagcctcctg

¶Genbank accession number (http://www.ncbi.nlm.nih.gov/).
